# Escaping from Air Pollution: Exploring the Psychological Mechanism behind the Emergence of Internal Migration Intention among Urban Residents

**DOI:** 10.3390/ijerph191912233

**Published:** 2022-09-27

**Authors:** Quan-Hoang Vuong, Tam-Tri Le, Quy Van Khuc, Quang-Loc Nguyen, Minh-Hoang Nguyen

**Affiliations:** 1 Centre for Interdisciplinary Social Research, Phenikaa University, Yen Nghia Ward, Ha Dong District, Hanoi 100803, Vietnam; 2 Faculty of Development Economics, VNU University of Economics and Business, Vietnam National University, Hanoi 100000, Vietnam; 3 SP Jain School of Global Management, Lidcombe, NSW 2141, Australia

**Keywords:** air pollution, migration intention, psychological mechanism, mindsponge theory, BMF analytics, urban development

## Abstract

Rapid urbanization with poor city planning has resulted in severe air pollution in urban areas of low- and middle-income countries. Given the adverse impacts of air pollution, citizens may develop ideation of averting behaviors, including migration to another region. The current study explores the psychological mechanism and demographic predictors of internal migration intention among urban people in Hanoi, Vietnam—one of the most polluted capital cities in the world. The Bayesian Mindsponge Framework (BMF) analytics was used to construct a model and perform Bayesian analysis on a stratified random sampling dataset of 475 urban people. We found that migration intention was negatively associated with an individual’s satisfaction with air quality. The association was moderated by the perceived availability of a nearby alternative (i.e., a nearby province/city with better air quality). The high migration cost due to geographical distance made the moderation effect of the perceived availability of a faraway alternative negligible. These results validate the proposed psychological mechanism behind the emergence of migration intention. Moreover, it was found that male and young people were more likely to migrate. While the brain drain effect did not clearly show, it is likely due to complex underlying interactions of various related factors (e.g., age and gender). The results hint that without air pollution mitigation measures, the dislocation of economic forces might occur and hinder sustainable urban development. Therefore, collaborative actions among levels of government, with the environmental semi-conducting principle at heart, are recommended to reduce air pollution.

## 1. Introduction

In recent decades, rapid urbanization with poor city planning has caused air pollution to become a serious problem affecting many people in cities worldwide. The World Health Organization (WHO) estimates that air pollution is responsible for about seven million total deaths yearly, with 4.2 million deaths due to ambient air pollution. Low- and middle-income countries suffer the highest level of exposure [1]. To confront air pollution, people have taken many adaptive strategies, such as reducing outdoor activities [2], increasing pharmaceutical purchases and medication usage [3], and particulate-filtering wearing facemasks [4]. In addition to these responses, migration to another city is a potential alternative when air pollution is inevitable. The current study’s purpose, thus, is to explore the psychological mechanism and demographic predictors of the internal migration intention of urban people using a dataset from Hanoi, Vietnam—one of the most polluted capital cities in the world [5].

Air pollution has negative impacts on, not only people’s health, but also their various psychological, economic, and social aspects [6]. Regarding mental health, exposure to air pollution is associated with general psychological distress [7], depressive disorder [8,9], and even suicide [10,11,12]. It is also worth noting that the subjective perception of air pollution and health risk can also affect annoyance and health symptoms even at non-toxic exposure levels [13]. Regarding impacts on human cognition, studies have shown that urban air pollution negatively affects children’s cognitive development [14,15] and their academic performance [16]. Air pollution also impairs cognitive function in adults [17,18] and especially in the elderly, which is a risk for developing dementia [19,20]. Studies have consistently found that air pollution reduces productivity in physical laborers [21] and white-collar workers [22].

Vietnam is a developing country that has one of the lowest air quality levels in the Asia-Pacific region. According to the 2022 Environmental Performance Index [23], Vietnam is ranked 18 out of 25 Asia-Pacific countries in the Air Quality category. Hanoi—the capital city of Vietnam—was ranked the world’s seventh most polluted capital in 2019, with an average PM_2.5_ level of 46.9 µg/m^3^ [24]. One of the main air pollution sources in Vietnam’s urban areas is traffic [25]. An assessment of the health risk induced by mobility in Hanoi shows that 3200 deaths can result from PM_10_ emitted by traffic [26]. Luong, et al. [27], using the data on daily admissions from Vietnam National Hospital of Pediatrics and daily records of air pollution, found that the increasing levels of particulate matter (such as PM_10_, PM_2.5,_ or PM_1_) are positively associated with respiratory admissions of young children in Hanoi. Also, exposure to smaller PM can lead to higher risk [27]. Moreover, air pollution in Vietnam urban areas is positively associated with cardiorespiratory hospitalizations [28] and pneumonia-related hospitalizations [29].

At the individual level, to protect themselves from the health risks of air pollution, people living in polluted urban areas may have the defensive response of averting behavior, meaning they limit exposure by lessening their time outdoors [30]. As examples of this coping strategy, higher air pollution levels are associated with a higher number of school absences [31], a lower level of outdoor cycling activities [32], and less usage of public parks [33].

In addition, environmental stress induced by air pollution can lead to migration as a response [34]. On national scales, internal migration from provinces or regions with worse air quality to those with higher air quality were observed, such as in Iran [35], Italy [36], and China [37]. High levels of air pollution also negatively affect the immigration rate in California counties [38] and temporary employment migrants’ desire to stay in Chinese cities [39], leading to further loss of human capital in the local region. 

Many studies on this topic have been conducted using data in Iran, Italy, the United States, and especially China, but little is known about the situation in other developing countries with a high level of air pollution. Although several studies have examined air pollution’s adversities in Vietnam, air pollution-induced migration has been under researched. 

In addition, while past studies have provided valuable findings on the relationship between air pollution and migration behavior, certain aspects receive limited attention. Most studies have focused on macro-scale investigations that employed particulate matter measurements and recorded population flows and changes [35,36,37]. This approach faces difficulties when delving deeper into specific psychological aspects among migrants. Scientists have advocated that it is not possible to develop effective policy responses against environmental stressors without understanding the roles of individuals’ perceptions because the environmental stress-induced decisions are affected by people’s subjective views rather than the actual stresses that can be objectively measured by scientific methods [40,41].

Therefore, we attempt to use Bayesian Mindsponge Framework (BMF) analytics to study the psychological mechanism behind the emergence of internal migration intention among Vietnamese urban people due to air pollution. The study’s findings can help generate more insight into the psychological responses to this environmental stressor and contribute empirical evidence to the growing field of environmental migration [42]. Specifically, it explains the changing internal migration intention likelihood of urban residents through the lens of their subjective cost–benefit judgments involving satisfaction level toward current air quality, perceived availability of better migration options, and the distance towards the options. We also examine the associations between socio-demographic characteristics and internal migration intention due to air pollution to check potential differences in air pollution-induced migration intention likelihood across distinct groups of people. Bayesian analysis was employed on the dataset of 475 urban people to test the proposed psychological mechanism of migration intention and the associations between socio-demographic characteristics and internal migration intention. 

This study consists of six sections. The Section 1 introduces the background and literature regarding environmental stress-induced migration as well as the study’s objectives. The Section 2 employs the mindsponge mechanism to propose the psychological mechanism of migration intention and construct the models for later statistical analysis. Next, we describe the details of materials and methods used to analyze the constructed model and validate the postulated psychological mechanism. The Section 4 presents the computed estimates using Bayesian analysis. Finally, the results are discussed with other existing literature and concluded in the Section 5 and Section 6, respectively.

## 2. Theoretical Foundation

We employ the mindsponge mechanism to formulate models to study the psychological mechanism behind pollution-induced internal migration intention. The mindsponge mechanism, with its complexity and dynamics, can be a good alternative for explaining the mental process that leads to the emergence of migration intention [43,44]. The mechanism assumes that each person has a mindset (a set of highly trusted values or beliefs) that shapes the value system and influences the person’s information processing mechanism within the mind (or multi-filtering process). The mechanism is a non-stop, continual absorption and ejection process of information that aims to maximize the person’s perceived benefits and minimize the perceived costs. Following this way of thinking, the responses of 475 urban residents at the time of the survey were the outcomes of their previous mental processes. Therefore, the mindsponge-based justification here aims to reconstruct the respondents’ mental processes that facilitate the emergence of internal migration intention in their minds. More details of this method can be found in [45].

There are two fundamental conditions for thoughts to emerge and persist in one’s mindset (ideation): information availability (it exists) and favorable evaluation (it is deemed beneficial by oneself) [46,47]. In other words, a piece of information needs to exist to be absorbed and processed, and it needs to pass through the subjective cost–benefit evaluations of the multi-filtering system for the ideation to occur within an individual’s mindset and influence subsequent mental processes. 

**Figure 1:**
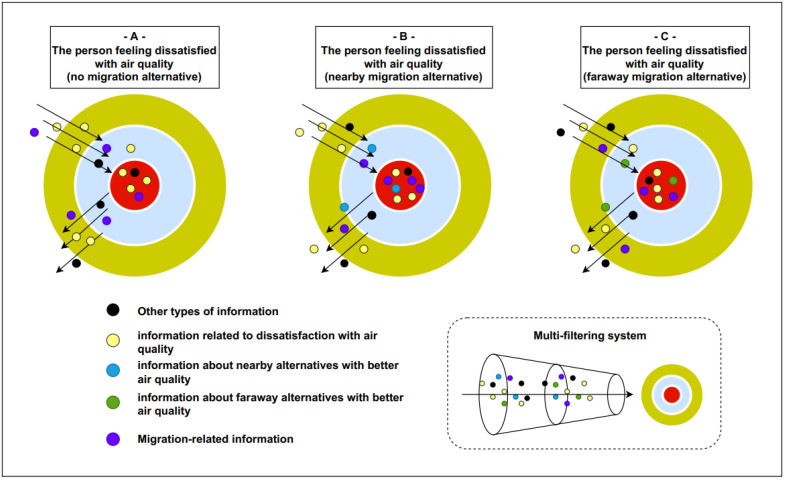
The information-based psychological mechanism leading to the emergence of migration intention.

Intention is generally defined as a prior conscious decision or a plan to perform a behavior (e.g., the definition of the American Psychological Association), reflecting both the notions of being formerly evaluated and determination. Based on the mindsponge mechanism described above, we assume that people with air pollution-induced migration intention have air pollution-induced migration-related information within their mindset. The information-based psychological mechanism leading to the emergence of migration intention is shown in Figure 1 for better clarity. In Figure 1, the red nucleus represents the mindset, the light-blue central circle represents the buffer zone (where the multi-filtering system kicks in to evaluate newly absorbed information from the environment), and the yellow outer circle represents the environment. In this study, we consider four main types of information: (1) migration-related information (purple particles), (2) information related to dissatisfaction with air quality (yellow particles), (3) information about nearby alternatives with better air quality (blue particles), and (4) information about faraway alternatives with better air quality (green particles). Other types of information are illustrated as black particles.

Objectively, air pollution can cause harm to human health and well-being. However, a person needs to perceive the harm to consider it, which affects subsequent mental processes. This perception can be proxied by a person’s feeling of satisfaction toward the air quality, which can reflect an approximation to the overall evaluation of air quality’s effects on their life (or experienced utility of air quality) [48,49]. If the person is dissatisfied, they are likely to experience negative consequences of air pollution and are more likely to absorb migration-related information into the mindset, and vice versa (see Scenario A in Figure 1). 

The perceived benefit of migrating is to escape from air pollution. Still, suppose the person does not know any places with better air quality. In that case, they are likely to perceive the risk of migrating to a place with similar or worse air quality compared to the origin city, which adds up to the net perceived cost of migrating. However, if a person perceives (or believes) a place with better air quality, this risk will be eliminated, reducing the net perceived cost of migrating. As a result, migration-related information is more likely to be accepted and persist in the mindset (see Scenarios B and C in Figure 1). 

Migrating can be perceived as costly in terms of both economic and psychic aspects. Regarding the economic aspect, a person needs to consider the availability of economic opportunities to assess the possibility of sustaining their life at the destination. However, due to the diminishing information with distance, they are less certain about the economic opportunities in faraway destinations, increasing the perceived cost of far-distance migration [50]. As for psychic cost, migrating to a faraway area with better air quality requires the person to leave familiar surroundings, adapt to a new environment, and acculturate to a new culture [50,51]. Because of the economic and psychic reasons, the person knowing faraway alternatives with better quality might perceive higher migration costs, making them less likely to accept migration-related information to enter and persist in the mindset and form migrating intention than those knowing nearby alternatives (see Scenarios B and C in Figure 1). Notably, the effects of perceived availability of nearby and faraway alternatives are not exclusive but can be additive to each other. In a sense, a person can perceive both nearby and faraway alternatives simultaneously, making them more likely to have migration intention than those perceiving either or none. 

From the proposed psychological mechanism of air pollution-induced migration intention above, it is plausible to say that there are three main factors influencing the emergence of migration intention: (1) the level of air quality satisfaction, (2) the perceived availability of options with better air quality, and (3) the consideration of moving distance. Model 1 is constructed to test whether our postulations are valid (see Table 1 for more details of variables):

(1) MigratIntention ~ α+AirSatisfaction+AirSatisfaction×NearbyMigratOpt+AirSatisfaction×FarawayMigratOpt 

Specifically, our proposed psychological mechanism is deemed valid if three conditions are met.

1. First, the association between *AirSatisfaction* and *MigratIntention* needs to be negative.
2. Second, the association between *AirSatisfaction* and *MigratIntention* has to be intensified (or positively moderated) by the perceived availability of options with better air quality. Here, we determined to treat *NearbyMigratOpt* and *FarawayMigratOpt* variables as moderating variables to turn their effects into non-linear (moderation) to avoid multicollinearity and confounding problems among predictor variables. We intentionally exclude the linear terms of *MigratIntention* with *NearbyMigratOpt* and *FarawayMigratOpt* because the model without them fits that data better than the model adding them (see Supplementary S1 for detailed comparison). Furthermore, excluding those linear terms can make the estimated results more understandable.
3. Third, to evaluate the effect of moving distance, we compare the moderation effects of *NearbyMigratOpt* and *FarawayMigratOpt*. If the moderation effect of *FarawayMigratOpt* is smaller than that of *NearbyMigratOpt*, our assumption that migration distance increases the perceived cost of migration, leading to lower migration likelihood, is valid. If their effects are equal or the effect of *NearbyMigratOpt* is smaller, our assumption will be invalid, as will our proposed psychological mechanism.

In the second model, we add the socio-demographic factors (age, gender, and education) into the model to examine their associations with migration intention. Doing so has two advantages. First, it helps identify potential migrants’ socio-demographic characteristics, which can generate insight for policy implications. Second, it tests the robustness of the results estimated by the first model. The second model is constructed as follows.

(2) MigratIntention ~ α+AgeGroup+Gender+Education+AirSatisfaction+AirSatisfaction×NearbyMigratOpt+AirSatisfaction×FarawayMigratOpt 

## 3. Materials and Methods

### 3.1. Materials

The data used in this study were retrieved from two open datasets about the perceptions of air pollution among inhabitants of Hanoi [52,53]. These datasets are the results of two survey collections using stratified random sampling methods conducted in the central and suburban areas of the city, respectively. The data were collected during November and December 2019. Hanoi was chosen as the study site due to three reasons: (1) Hanoi was ranked 7th among the most polluted capital cities around the world [5]; (2) Hanoi is a fast-growing city in Vietnam; and (3) Hanoi is the second largest and most populous city in Vietnam. 

Normally, migrants are attracted to growing big cities for better job opportunities [54,55]. However, the accumulation of anthropogenic activities in such cities (e.g., traffic, construction) can result in air pollution, which can possibly lead to averting behaviors and intentions through internal migration. Hanoi’s city features make it representative of other urban areas, not only in Vietnam, but also in other developing countries with similar socio-demographic characteristics for studying the underlying psychological mechanism of internal migration.

According to Khuc, Phu and Luu [53], the survey collection consisted of three steps. First, the collectors were recruited and paid to encourage them to perform well during the collection process. The researchers also held two four-hour seminars to help the collectors understand the project’s goals and the questionnaire’s content. During the seminar, necessary skills and techniques to extract information from respondents were also discussed. Then, two pilot tests were conducted to ensure the final version was error-free, straightforward, and easy to understand. Lastly, the collectors conducted face-to-face interviews with the respondents and maintained mutual interaction and communication to solve issues or answer questions arising during the survey collection. There was a total of 475 respondents, of which the majority were in the age group of 19–40 (52.84%). Male respondents accounted for 54.53% of the total respondents, while female respondents accounted for 45.26%. Among 475 respondents, approximately 10% reported their intention to move to another province/city to live and work due to air pollution in their current places. See Table A1 in the Appendix A for more descriptive statistics.

Following the conceptual models explained in the Theoretical Foundation section, we generated seven variables to be used for Bayesian analysis: six predictor variables and one outcome variable (see Table 1).

The outcome variable is *MigratIntention*, created from the question, “Do you intend to move your family and work in another province/city with less pollution?” Two answers are possible: ‘yes’ and ‘no’. 

The urban people’s satisfaction with the current air quality level is determined by asking, “Overall, how satisfied are you with the air quality?”, and demonstrated by the *AirSatisfaction* variable. The air satisfaction level is rated on a four-point Likert scale ranging from one (‘very dissatisfied’) to four (‘very satisfied’).

*NearbyMigratOpt* and *FarawayMigratOpt* variables were modified from two original items in the dataset. Originally, Khuc, Phu and Luu [53] asked the respondents, “How do you feel about the air quality in Hanoi compared to neighboring provinces/cities?” and “How do you feel about the air quality in Hanoi compared to southern provinces/cities?” Four answers were possible: ‘better than’, ‘same as’, ‘less than’, and ‘I don’t know’. 

While the *NearbyMigratOpt* variable is an unambiguous indication of nearby availability of provinces/cities with better air quality, the variable, *FarawayMigratOpt,* needs some contextual knowledge to comprehend. Specifically, Ho Chi Minh City and Hanoi are the two largest cities in Vietnam. While Hanoi is the capital city in North Vietnam, Ho Chi Minh is the largest city in the South, which is 1137 km away from Hanoi. In Vietnam, people usually use ‘the North’ to indicate Hanoi and its nearby provinces/cities, and ‘the South’ to indicate Ho Chi Minh city and its surrounding provinces/cities [56,57]. Despite being in the same country, these two regions have some distinct cultural characteristics due to the complex cultural change and historical events [58]. Given the distance and some socio-cultural differences between Hanoi and the South of Vietnam, it is plausible to use the variable *FarawayMigratOpt* to represent the perceived faraway options with better air quality. In addition, for investigating the impact of perceived option availability, modifications were made to turn them into binary variables, with one being ‘less than’, and zero being ‘better than’ and ‘same as’. The respondents that reported ‘I don’t know’ were excluded from the analysis.

### 3.2. Methods and Validation

The Bayesian Mindsponge Framework (BMF) analytics was employed to construct and analyze models that support the examination of internal migration induced by air pollution [59]. Specifically, we constructed three models based on the mindsponge framework of information processing [43,44] and performed Bayesian analysis to examine the constructed models. The analytical framework has been effectively applied in investigating the psychological mechanisms underneath human thinking and behaviors [46,60,61,62,63]. 

There are five reasons that Bayesian analysis was employed in the current study. First of all, science is now facing the reproducibility crisis that a large proportion of studies across disciplines cannot be replicated. Psychology [64] and social sciences [65] are not excluded. One of the main reasons is the wide sample-to-sample variability in the *p*-value. Bayesian inference can be a good alternative for the *p*-value approach employed in frequentist inference, as estimation and visualization of the credible intervals are basic features of Bayesian analysis [66]. 

Secondly, Bayesian inference treats all the properties probabilistically, including unknown parameters, so it has high compatibility with the current study’s design, which is explanatory in nature (employing the mindsponge framework to explain the underlying psychological mechanism of migration intention). By treating all properties probabilistically, the Bayesian analysis helps us consider the impacts of other unknown factors while maintaining the rule of parsimoniousness for the explanation [67].

Our models examined the moderation effects of *NearbyMigratOpt* and *FarawayMigratOpt* on the relationship between *MigratIntention* and *AirSatisfaction*. These non-linear terms made the model more complex and would require a large sample size for sound estimation [68]. The Bayesian analysis integrating the Markov Chain Monte Carlo (MCMC) technique generates a large number of parameters’ samples through stochastic processes of Markov chains, so it can help fit complex models effectively [69]. 

Moreover, Bayesian analysis does not rely on asymptotics, which is a hindrance for frequentist methods in estimating small sample size datasets, so it could provide a more precise estimation for a small sample size dataset by incorporating the appropriate prior distributions [70]. The prior distributions in this study were identified and rationalized based on the mindsponge mechanism.

Finally, prior distribution incorporation is another advantage of Bayesian analysis, especially when being employed with the mindsponge mechanism, because it allows researchers to incorporate prior knowledge and theoretical formulation into the statistical estimation. Also, setting informative priors before fitting models can alleviate the risk of multicollinearity because it helps solve the weak data identification problems [71,72,73]. Some scientists may criticize prior incorporation because of subjectivity bias. However, such bias can be reduced because our priors were identified and rationalized based on the mindsponge mechanism. Moreover, to deal with this criticism, we also performed the “prior-tweaking” technique [74]. The technique is a way to check the model’s robustness. Prior-tweaking is to recompute the posterior estimates using informative priors reflecting our disbelief in the proposed associations. If the estimated posteriors’ effect tendencies using priors reflecting our belief in the effects are not different from those estimated using priors reflecting our disbelief in the effects, the models’ results can be deemed robust without bias over the existence of the effects. 

For validating the simulated posterior outcomes, we adopted a four-pronged validation strategy. Initially, we conducted a goodness-of-fit check on each simulated model using the PSIS-LOO diagnostic plots [75]. If the *k* values shown on the plot are all below 0.5, the model can be deemed a good fit with the data. Next, we checked the Markov chains’ convergence using the diagnostic statistics and plots. The statistics include the effective sample size (*n_eff*) and the Gelman shrink factor (*Rhat*), while the plots include the trace plots, Gelman plots, and autocorrelation plots. Then, the prior-tweaking technique was performed to confirm the models’ robustness. Finally, we compared the weight between Models 1 and 2 to check which model had better predictive accuracy over the data. The model with better predictive accuracy was used for discussion and computing the probabilities of migration intention among urban residents. Further explanation and interpretation of the diagnostic statistics, plots, prior-tweaking technique, weight comparison, and probability calculation are presented in the Results section.

The **bayesvl** R package was used to perform Bayesian analysis in the current study for three reasons: (1) it is a cost-effective alternative; (2) it has a great capability to visualize eye-catching graphics; and (3) it is easy to operate [76,77]. Bayesian analysis aided by MCMC simulation was performed with 5000 iterations, 2000 warm-up iterations, and four Markov chains to estimate Models 1 and 2. The dataset, data description, and code snippets of the Bayesian analysis were deposited in the Open Science Framework repository (https://osf.io/us5tr/ (accessed on 3 July 2022)).

## 4. Results

### 4.1. Model 1: Migration Cost–Benefit Judgment

**Figure 2:**
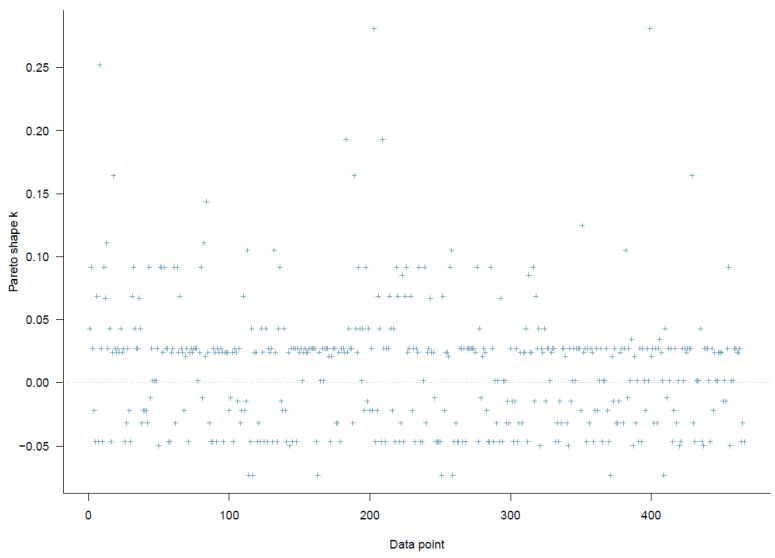
Model 1′s PSIS diagnostic plot.

The first model examined the effects of citizens’ satisfaction with air quality and its interactions with perceived better nearby and faraway alternatives on migration intention. The PSIS diagnostic plot shows that all *k* values are below 0.3, suggesting that Model 1 has a high goodness-of-fit with the data (see Figure 2). 

The effective sample size (*n_eff* > 1000) and Gelman shrink factor (*Rhat* = 1) of all simulated posterior coefficients portray a good convergence of the model’s Markov chains (see Table 2). The convergence can also be visually diagnosed using the trace plots, autocorrelation plots, and Gelman plots.

**Figure 3:**
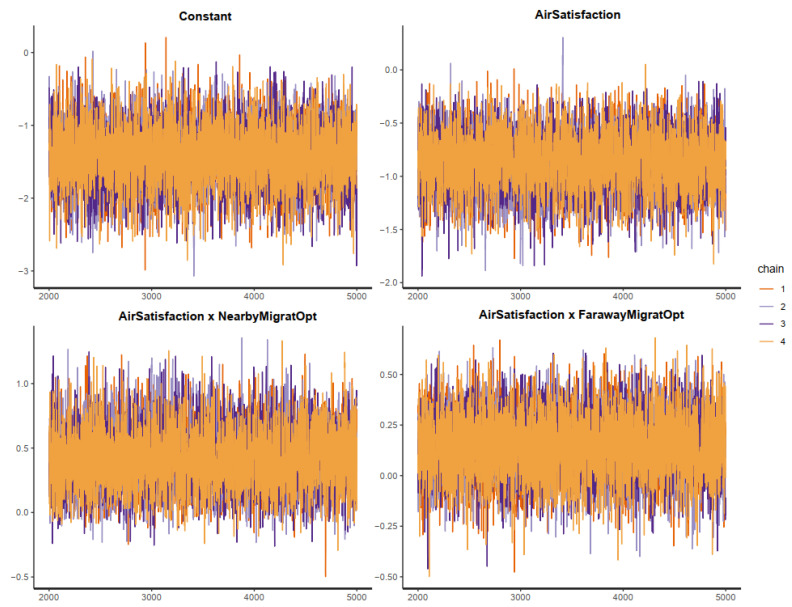
Trace plots for Model 1′s posterior parameters.

Figure 3 demonstrates the trace plots of all posterior parameters. The *y*-axis of the trace plots represents the posterior values of each parameter, while the *x*-axis represents the iteration order of the simulation. The colored lines in the middle of the trace plots are Markov chains. If the Markov chains fluctuate around a central equilibrium, they can be considered good-mixing and stationary. These two characteristics are a good signal of convergence. More convergence diagnoses using the Gelman and autocorrelation plots are available in Supplementary S2 (see Figures S1 and S2).

From the simulated posterior results of Model 1, we found that the citizens’ satisfaction with air quality was negatively associated with the intention to migrate to another province/city ($\mu_{AirSatisfaction}=-0.86$ and $\sigma_{AirSatisfaction}=0.27$). This result confirms our assumption that more satisfied people are less likely to migrate due to the lower perceived benefit of leaving the city. However, the cost–benefit judgment of urban citizens about migration due to air pollution is much more complex. Perceiving alternatives with better air quality (either nearby provinces/cities or faraway provinces/cities in the South) moderated the effect of air satisfaction on migration intention ($\mu_{AirSatisfaction\;\times \;NearbyMigratOpt}=0.42$ and $\sigma_{AirSatisfaction\;\times \;NearbyMigratOpt}=0.23$; $\mu_{AirSatisfaction\;\times \;FarawayMigratOpt}=0.15$ and $\sigma_{AirSatisfaction\;\times \;FarawayMigratOpt}=0.16$). The moderation impact of perceiving faraway alternatives is smaller than the nearby alternatives, meaning that people who are not satisfied with the current air quality will be more likely to move to a province/city nearby rather than a faraway province/city (in this case the Southern area). These results validate our assumptions on the moderation effect of the perceived availability of alternatives with better air quality and the effect of migration distance, respectively. 

For robustness check, prior-tweaking was performed using informative priors that reflected our disbelief in the predictions. In both cases (priors representing our belief and disbelief on the predictions), the coefficients’ effect patterns are similar, although the effect degree slightly changes (see Table 2). We can conclude that the effects in Model 1 are robust even when the priors vary. 

**Figure 4:**
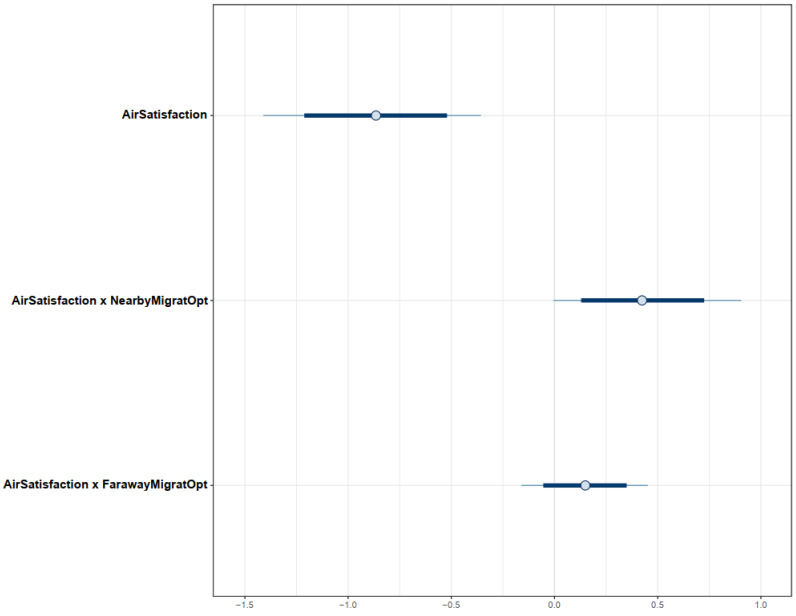
Distributions of Model 1’s posterior coefficients on an interval plot.

The distributions of Model 1′s parameters are visualized in the interval plot (see Figure 4) to assess their reliability. The *x*-axis of the interval plot demonstrates the posterior values of parameters. The distribution of the coefficient *AirSatisfaction* lies entirely on the negative side of the axis, indicating a highly reliable negative association between *AirSatisfaction* and *MigratIntention*. Distributions of coefficients *AirSatisfaction × NearbyMigratOpt* and *AirSatisfaction × FarawayMigratOpt* are mostly located on the positive side, implying that the moderation effects of perceived availability of alternatives with better air quality have the highest probability of being positive. 

**Figure 5:**
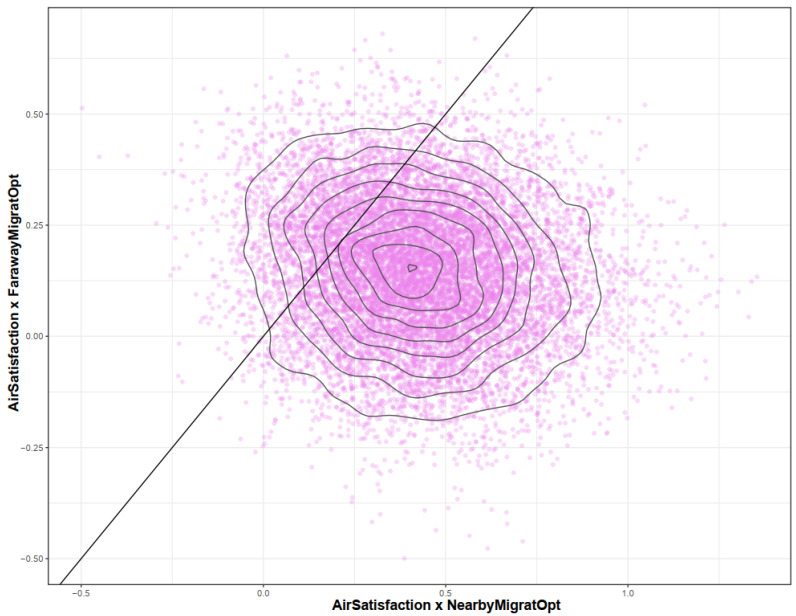
Pairwise distribution plot for Model 1′s *AirSatisfaction* × *NearbyMigratOpt* and *AirSatisfaction* × *FarawayMigratOpt*.

It is shown in Figure 5 that *AirSatisfaction* × *NearbyMigratOpt* has a greater moderation effect than *AirSatisfaction* × *FarawayMigratOpt*. Specifically, a higher proportion of simulated samples have positive values according to the *x*-axis than the *y*-axis, so the positive moderation effect of *AirSatisfaction* × *NearbyMigratOpt* can be deemed to be greater and more reliable than *AirSatisfaction* × *FarawayMigratOpt*. This result confirms our assumption on the role of migration distance in the psychological mechanism of air pollution-induced migration intention.

### 4.2. Model 2: Incorporation of Socio-Demographic Factors

**Figure 6:**
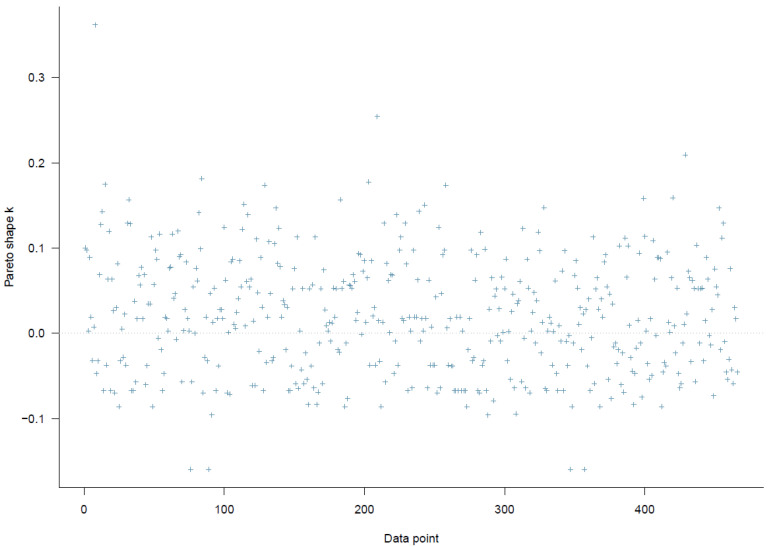
Model 2′s PSIS diagnostic plot.

The second model incorporates socio-demographic factors (e.g., age, gender, educational level) into Model 1 to test the robustness of the results presented above and identify potential migrants’ characteristics. Similar to Model 1, the PSIS diagnostic plot of Model 2, illustrated in Figure 6, indicates that the model has a high goodness-of-fit with the data (*k* < 0.4). 

**Figure 7:**
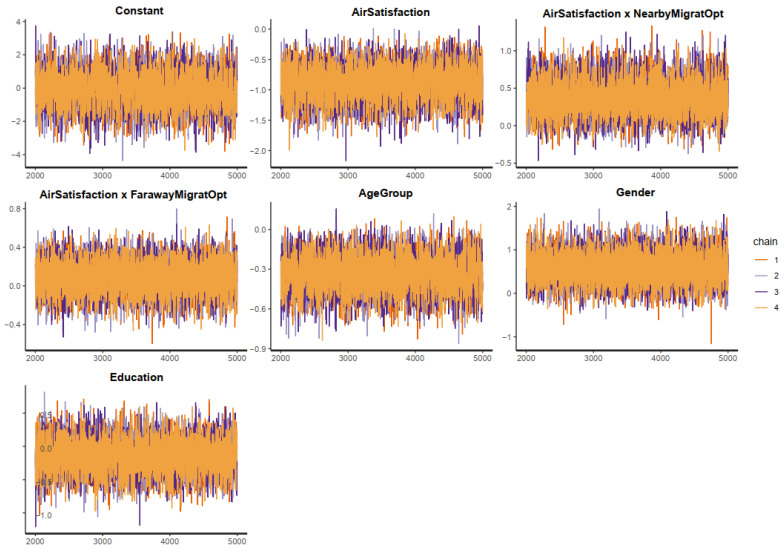
Trace plots for Model 2′s posterior parameters.

The trace plots for Model 2′s posterior parameters show “clean and healthy” Markov chains (see Figure 7). Moreover, the Gelman and autocorrelation plots (see Figures S3 and S4) also demonstrate a good convergence signal. As a result, the Markov chain central limit theorem holds in Model 2′s simulation. The diagnostic statistics also confirm this statement, as all the *n_eff* values are greater than 1000, and *Rhat* values are equal to one (see Table 3).

**Figure 8:**
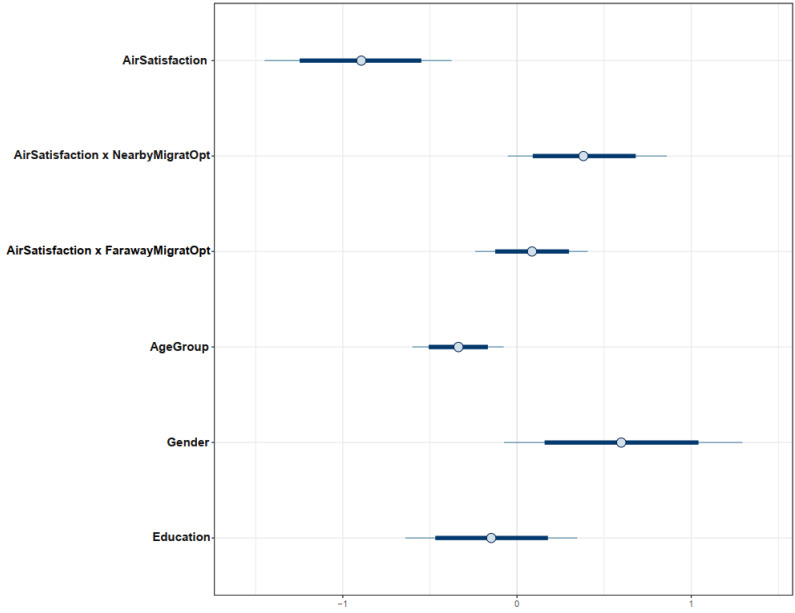
Distributions of Model 2′s posterior coefficients on an interval plot.

The effects of *AirSatisfaction* and its interaction terms estimated in Model 2 are consistent with those in Model 1. In particular, *AirSatisfaction* is negatively associated with *MigratIntention* ($\mu_{AirSatisfaction}=-0.89$ and $\sigma_{AirSatisfaction}=0.27$). Even though the moderation effect of *FarawayMigratOpt* on the relationship between *AirSatisfaction* and *MigratIntention* is negligible ($\mu_{AirSatisfaction\;\times \;FarawayMigratOpt}=0.09$ and $\sigma_{AirSatisfaction\;\times \;FarawayMigratOpt}=0.17$), it validates our assumption on the impact of distance on the citizens’ migration cost–benefit judgment because it still has a positive association, and its moderation effect is weaker than that of *AirSatisfaction*×*NearbyMigratOpt* ($\mu_{AirSatisfaction\;\times \;NearbyMigratOpt}=0.38$ and $\sigma_{AirSatisfaction\;\times \;NearbyMigratOpt}=0.23$). We also found no significant changes in the simulated posterior results using the prior-tweaking technique, except for the declining coefficients’ effect degree (see Table 3). All coefficients’ distributions are presented in Figure 8.

### 4.3. Model Comparison and Migration Probability Visualization

We employed four different values for comparing Models 1 and 2: WAIC, Pseudo-BMA without Bayesian bootstrap, Pseudo-BMA with Bayesian bootstrap, and Bayesian stacking (see Table 4). The Akaike weight was also used to rescale these values [78]. A total weight of one is partitioned among the two models, with the greater-weight model implying better predictive accuracy on the data. As can be seen from Table 4, Model 2 has the highest weight in all categories, so we can say that Model 2 has better predictive accuracy over the data than Model 1 and should be used for later probability calculation of migration intention and discussion.

Based on the simulated posterior coefficients of Model 2 (see Table 3), we can use the logit model below to calculate the probability of citizens’ migration intention. More details of the logit model are provided by Penn State at the following website: https://online.stat.psu.edu/stat504/lesson/6 (accessed on 23 September 2022). The parameters’ mean values should be used because they indicate the highest probability of happening.

(3) $$\begin{array}{ll} \ln\frac{\pi_{migration}}{\pi_{no\;migration}} & =-0.11-0.34\times AgeGroup+0.60\times Gender-0.15\times Education-0.89\times AirSatisfaction\\ & +0.38\times AirSatisfaction\times NearbyMigratOpt\\ & +0.09\times AirSatisfaction\times FarawayMigratOpt \end{array}$$

Based on this model, for example, we can calculate the migration intention probability of female citizens in the age group of 31–40, with an educational level of college/university, being dissatisfied with the current living place’s air quality, and perceiving nearby provinces/cities with better air quality, but not one that is far away in the South of Vietnam.

(4) $$\pi_{migration}=\frac{e^{-0.11\;-\;0.34\;\times \;3\;+\;0.60\;\times \;0\;-\;0.15\;\times \;3\;-\;0.89\;\times \;2\;+\;0.38\;\times \;1\;\times \;2\;+\;0.09\;\times \;0\;\times \;2}}{1+e^{-0.11\;-\;0.34\;\times \;3\;+\;0.60\;\times \;0\;-\;0.15\;\times \;3\;-\;0.89\;\times \;2\;+\;0.38\;\times \;1\;\times \;2\;+\;0.09\;\times \;0\;\times \;2}}=0.0691=6.91\%$$

**Figure 9:**
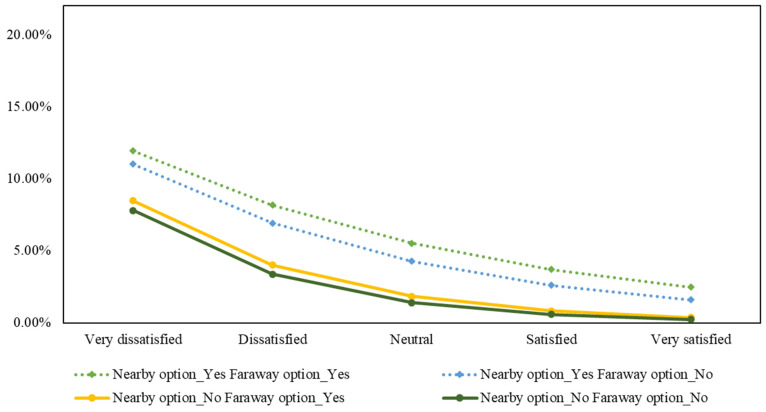
Probabilities of migration intention in female citizens, aged 31–40, college level, based on air quality satisfaction, perceived availability of nearby and faraway migration alternatives.

**Figure 10:**
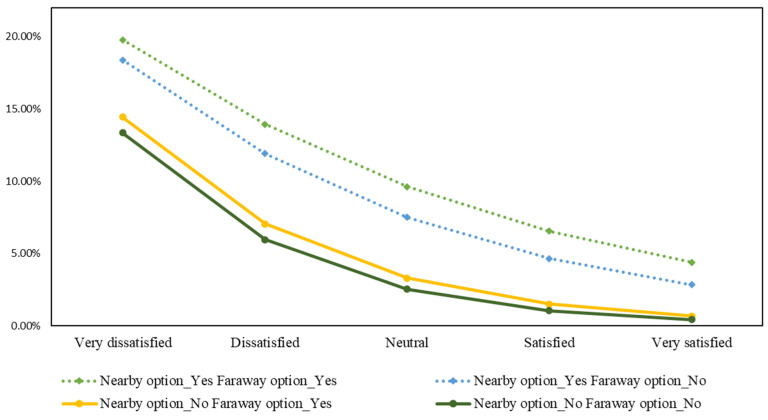
Probabilities of migration intention in male citizens, aged 31–40, college level, based on air quality satisfaction, perceived availability of nearby and faraway migration alternatives.

Likewise, the migration intention probabilities of other scenarios are calculated and visually compared in Figure 9. Applying a similar calculation method, we also visualize the probabilities of migration intention in male citizens aged 31–40, college level, based on air quality satisfaction, perceived availability of nearby and faraway migration alternatives in Figure 10.

As seen from both Figure 9 and Figure 10, the highest probability of migration intention occurs when the person is very dissatisfied with the current air quality in Hanoi and simultaneously perceives nearby and faraway alternatives with better air quality. While perceiving nearby alternatives with better air quality has a clear impact on the migration probabilities across different levels of air satisfaction, the effect of perceiving faraway alternatives is minimal, especially when the person is very satisfied with the current air quality and perceives no nearby options. This visualization confirms our assumptions that residents’ migration intention probabilities are influenced by their subjective cost–benefit judgements involving satisfaction with current air quality, perceived availability of better migration options, and the distance toward the options. 

## 5. Discussion

The current study explores the psychological mechanism behind migration intention due to air pollution by employing BMF analytics on the responses of 475 urban people participating in a stratified random sampling survey in Hanoi—one of the most polluted capital cities in the world. Several major findings were identified from the simulated posterior results.

First, our results show that the intention to migrate to another living place was negatively associated with the level of satisfaction with the current environment’s air quality (see Model 1). In other words, people who dislike the polluted air in their city are more likely to have an intention to move away. This is consistent with findings reported in previous studies using macro-indicators investigating the relationship between air pollution and internal migration behavior [35,36,37]. Moreover, our results support the idea that people may develop the intention of migrating away from their current living areas with high pollution levels as an averting strategy to reduce environmental health risks [34]. This specific type of deliberate risk avoidance thinking can be elaborated in what follows. 

Specifically, there are two fundamental conditions for a value to be accepted into the mindset: information availability/accessibility and a positive evaluation of the cost–benefit judgment. Thus, the migration intention to another province/city can be deemed to appear in an individual’s mindset when an individual perceives it to be beneficial. Intuitively, the information about environmental health risks induced by poor air quality holds a clear negative value on the cost–benefit scale (in other words, living in a polluted place is subjectively perceived as “costly”). This, in turn, adds more benefit to migration and increases the possibility of the migration idea being accepted into the mindset.

The cost–benefit judgment process of whether to move is multiplex. Besides the satisfaction level with the air quality, it also depends on many factors, including the availability of better choices and the distance from the home city to the destination. We found that both the perceived availability of a better nearby alternative and a better faraway alternative moderated the effect of air satisfaction on migration intention. However, the moderation effect of the perceived availability of a better faraway alternative was minimal. This finding confirms our assumption on the role of migration distance in the subjective cost-benefit consideration of urban residents. Besides financial cost (e.g., traveling expenditure), increasing psychic cost and information-diminishing might contribute to the negative effect of long distance on migration intention [50,79]. The farther the destination is, the less available information a person has about job opportunities, social networks, living conditions, and social norms at the destination. This is not to say that the differences in cultural values between northern and southern regions in Vietnam also exist. These factors significantly increase the perceived risk of moving. 

Demographic factors are also important predictors of migration intention. Our results showed that younger people (compared to older ones) and males (compared to females) were more likely to have migration intentions due to air pollution. Older people may perceive more difficulties migrating due to issues related to older age, such as job opportunities or health conditions. Regarding gender, Vietnamese women often prioritize taking care of children and other family members, and these connections may cause the decision to migrate to be more costly for females. It is also suggested that females may be more risk averse than males, so they might perceive the decision to migrate as riskier and are less likely to have migration intention [80,81,82]. Nevertheless, we should not firmly overgeneralize the influences of age and gender on migration behaviors because they are very complex and situation-specific [83]. Further studies on these factors are necessary to explore them more deeply.

Regarding the effect of educational level, the results in the present study do not show statistical significance. However, the statistical insignificance of the present study’s models is due to the complex interactions of many underlying factors that are associated with age and gender, as supported by the findings of a study using the same dataset focusing more deeply on the brain drain effect [62]. 

The psychological mechanism of migration intention indicates that pollution-induced internal migration, especially among young and male populations, is probable and could be detrimental to local development. If the air pollution level is not reduced, public awareness of air quality will rise [84] and affect migration choice [39]. Eventually, the internal migration induced by poor air quality might lead to the relocation of economic forces (especially male and young people with high educational levels) and hinder sustainable development in the origin city. Khuc, Nguyen, Le, Nguyen, Nguyen, Lich and Vuong [62] find that young males (below 40 years old) in Hanoi have a probability of pollution-induced internal migration from nearly 10% to over 30%, depending on their educational levels.

Thus, collaborative actions among levels of government are required to mitigate air pollution from the main sources of urban air pollution in Vietnam, like traffic, industrial emissions, and construction sites [25,85]. Although the Vietnamese government issued the *National action plan on air quality management by 2020 with the vision to 2025* (Decision No 9851/QD-TTG) to reduce air pollution, its effectiveness remained contentious [86]. Therefore, the national government should strengthen its air quality monitoring and evaluation systems and provide more legislative and financial support to the municipal government. On the side of municipal administrations, they need to take the responsibility to provide support and enforce strict environmental regulations toward the private sector and improve urban planning quality and public communication regarding air pollution [74,87]. Furthermore, Vietnam’s governmental support should focus on scientific research, which helps set effective and efficient courses of action [88]. For air pollution to be effectively and efficiently reduced, the semi-conducting principle, suggesting that monetary values cannot be used interchangeably for environmental values, should be employed as the core ideology when designing, planning, and implementing mitigation measures [89].

Limitations of the current study are described here for transparency [90]. The current study only employed data from the capital city of Hanoi, so the findings regarding associations between demographic factors and migration intention might be restricted to the socio-cultural characteristics of the study site. However, the investigated cost-benefit judgment can be deemed a natural thinking mechanism, so the socio-cultural differences might not be significant, and the study’s findings can be generalized, to some extent, to other cities within Vietnam and other developing countries. Moreover, there is a certain gap between ideation and behavior, so migration intentions should be interpreted as a potential action rather than actual action. Future studies are recommended to examine how much the migration intention is associated with actual migration behavior, or validate whether the subjective cost–benefit judgments shown in our study influence the actual migration behavior to fill in this shortage. Migration destination is not only limited to domestic cities or provinces but also include foreign cities or regions, so exploring international migration intention/behavior induced by air pollution is a promising research topic for future studies.

## 6. Conclusions

Due to dissatisfaction with the current air quality, citizens may have the intention of moving away as a risk avoidance behavior. Based on the mindsponge framework of information processing, we point out some major aspects of the psychological mechanism underlying such intention: (1) the satisfaction level of current living place’s air quality, (2) perceived availability of alternatives with better air quality, and (3) the consideration of moving distance. We found that more dissatisfied people are more likely to have migration intentions. Perceived availability of provinces/cities nearby as alternative living places strengthens this effect. Perceived availability of better faraway alternatives also does, but only with a minimal degree. The findings validate our proposed psychological mechanism, which shows that subjective cost–benefit evaluation greatly influences air pollution-induced migration intention. Additionally, we found that male and young people tend to have higher probabilities of migration intention, but more specific studies on demographic characteristics are needed to examine the effects accurately. Based on the findings, we suggest the government carefully consider this psychological mechanism for making integral urban planning and sustainability policies.

## Figures and Tables

**Table 1:** Variable description.

Variable	Meaning	Type of Variable	Value
*MigratIntention*	Whether the respondent had the intention to immigrate to another province/city due to air pollution	Binary	Yes = 1 No = 0
*AirSatisfaction*	The respondent’s satisfaction level with the current air quality	Ordinal	From 1 (very dissatisfied) to 5 (very satisfied)
*Nearby* *MigratOpt*	Whether the respondent knows a better nearby option to migrate. In other words, whether the respondent perceived that neighboring provinces/cities had better air quality	Binary	Yes = 1 No = 0
*FarawayMigratOpt*	Whether the respondent knows a better faraway option to migrate. In other words, whether the respondent perceived that Southern provinces/cities had better air quality	Binary	Yes = 1 No = 0
*AgeGroup*	The age group that the respondent belongs to	Ordinal	From 10 to 18 = 1 From 19 to 30 = 2 From 31 to 40 = 3 From 41 to 50 = 4 From 51 to 60 = 5 Above 60 = 6
*Gender*	Gender	Binary	Male = 1 Female = 0
*Education*	The highest achieved level of education of the respondent	Ordinal	Secondary school or below = 1 High school = 2 Technical school, college degree, university degree = 3 Master’s degree = 4 Doctoral degree = 5

**Table 2:** Model 1′s simulated posteriors.

Parameters	Priors Reflecting Belief on Effects				Priors Reflecting Disbelief On Effects			
	Mean	SD	*n_eff*	*Rhat*	Mean	SD	*n_eff*	*Rhat*
*Constant*	−1.41	0.42	7081	1	−1.60	0.42	6532	1
*AirSatisfaction*	−0.86	0.27	5588	1	−0.47	0.24	5623	1
*AirSatisfaction × NearbyMigratOpt*	0.42	0.23	6662	1	0.13	0.19	6751	1
*AirSatisfaction × FarawayMigratOpt*	0.15	0.16	8217	1	0.07	0.16	8623	1

**Table 3:** Model 2′s simulated posterior coefficients.

Parameters	Priors Reflecting Belief on Effects				Priors Reflecting Disbelief on Effects			
	Mean	SD	*n_eff*	*Rhat*	Mean	SD	*n_eff*	*Rhat*
*Constant*	−0.11	1.06	4779	1	−0.45	1.03	4588	1
*AirSatisfaction*	−0.89	0.27	6712	1	−0.49	0.24	7273	1
*AirSatisfaction × NearbyMigratOpt*	0.38	0.23	7151	1	0.08	0.20	8332	1
*AirSatisfaction × FarawayMigratOpt*	0.09	0.17	10,652	1	0.00	0.16	12,045	1
*AgeGroup*	−0.34	0.13	6461	1	−0.34	0.13	7147	1
*Gender*	0.60	0.35	10,751	1	0.59	0.34	11,862	1
*Education*	−0.15	0.25	5810	1	−0.09	0.25	5923	1

**Table 4:** Weight comparison.

	WAIC	Pseudo-BMA without Bayesian Bootstrap	Pseudo-BMA with Bayesian Bootstrap	Bayesian Stacking
Model 1	0.13	0.13	0.31	0.33
Model 2	0.87	0.87	0.69	0.67

## Data Availability

The data and code that support the findings of this study are available on The Open Science Framework for later replication (https://osf.io/us5tr/ (accessed on 3 July 2022)).

## Supplementary Materials

The following supporting information can be downloaded at: https://www.mdpi.com/article/10.3390/ijerph191912233/s1, Supplementary S1: Comparison between Models 1a and 1b, Table S1: Posterior estimates of Model 1a using uninformative priors, Table S2: Posterior estimates of Model 1b using uninformative priors, Table S3: Weight comparison between Models 1a and 1b; Supplementary S2: Models’ convergence diagnoses, Figure S1: Gelman plots for Model 1’s posterior parameters, Figure S2: Autocorrelation plots for Model 1’s posterior parameters, Figure S3: Gelman plots for Model 2’s posterior parameters, Figure S4: Autocorrelation plots for Model 2’s posterior parameters.

## Appendix A

**Table A1:** Variables’ descriptive statistics.

Variable	Level	Samples (N = 475)	Proportion
*MigratIntention*	No = 0	430	90.53%
	Yes = 1	44	9.26%
*AirSatisfaction*	Very dissatisfied = 1	102	21.47%
	Dissatisfied = 2	237	49.89%
	Neutral = 3	111	23.37%
	Satisfied = 4	20	4.21%
	Very satisfied = 5	2	0.42%
*NearbyMigratOpt*	No = 0	85	17.89%
	Yes = 1	390	82.11%
*FarawayMigratOpt*	No = 0	323	68.00%
	Yes = 1	152	32.00%
*AgeGroup*	From 10 to 18 = 1	8	1.68%
	From 19 to 30 = 2	142	29.89%
	From 31 to 40 = 3	109	22.95%
	From 41 to 50 = 4	63	13.26%
	From 51 to 60 = 5	61	12.84%
	Above 60 = 6	92	19.37%
*Gender*	Female = 0	215	45.26%
	Male = 1	259	54.53%
*Education*	Secondary school or below = 1	53	11.16%
	High school = 2	132	27.79%
	Technical school, college degree, university degree = 3	265	55.79%
	Master’s degree = 4	19	4.00%
	Doctoral degree = 5	2	0.42%
