# Supplementary material for: Escaping from Air Pollution: Exploring the Psychological Mechanism behind the Emergence of Internal Migration Intention among Urban Residents
Source: Int J Environ Res Public Health. 2022 Sep 27;19(19):12233. doi: 10.3390/ijerph191912233 (PMC9566140; doi:10.3390/ijerph191912233)
Supplement: Supplementary file 1 [file ijerph-19-12233-s001.zip › Supplementary S1_Model comparison.pdf]

## Supplementary S1: Comparison between Models 1a and 1b

In this Supplementary, we present the weight comparison between Models 1a and 1b to determine whether the inclusion of the linear relationship of *MigratIntention* with *NearbyMigratOpt* and *FarawayMigratOpt* makes the model more well-specified than no inclusion. Model 1a presented below only includes the non-linear relationships of *MigratIntention* with *NearbyMigratOpt* and *FarawayMigratOpt*, whereas Model 1b includes both the linear and non-linear relationships of *MigratIntention* with *NearbyMigratOpt* and *FarawayMigratOpt*.

**Model 1a:** $MigratIntention \sim \alpha + AirSatisfaction + AirSatisfaction * NearbyMigratOpt + AirSatisfaction * FarawayMigratOpt$

**Model 1b:** $MigratIntention \sim \alpha + AirSatisfaction + NearbyMigratOpt + AirSatisfaction * NearbyMigratOpt + FarawayMigratOpt + AirSatisfaction * FarawayMigratOpt$

After simulating both Models 1a and 1b using uninformative priors, we obtained the posterior estimates shown in Tables S1 and S2.

**Table S1:** Posterior estimates of Model 1a using uninformative priors

| Parameters | Mean | SD | n_eff | Rhat |
|---|---|---|---|---|
| *Constant* | -1.44 | 0.45 | 6397 | 1 |
| *AirSatisfaction* | -0.67 | 0.31 | 4424 | 1 |
| *AirSatisfaction* NearbyMigratOpt* | 0.26 | 0.25 | 6160 | 1 |
| *AirSatisfaction* FarawayMigratOpt* | 0.06 | 0.17 | 8012 | 1 |

**Table S2:** Posterior estimates of Model 1b using uninformative priors

| Parameters | Mean | SD | n_eff | Rhat |
|---|---|---|---|---|
| *Constant* | -2.52 | 1.19 | 2853 | 1 |
| *AirSatisfaction* | -0.25 | 0.54 | 2842 | 1 |
| *NearbyMigratOpt* | 0.47 | 1.26 | 2858 | 1 |
| *AirSatisfaction* NearbyMigratOpt* | 0.10 | 0.58 | 2841 | 1 |
| *FarawayMigratOpt* | 1.62 | 0.94 | 3795 | 1 |
| *AirSatisfaction* FarawayMigratOpt* | -0.72 | 0.49 | 3902 | 1 |

Comparatively, Model 1b parameters' effective sample size (n_eff) values are significantly lower than those of Model 1a, indicating that Model 1a has better convergence than Model 1b (see Table S1 and S2). This signals that Model 1a may fit Model 1b better. To reconfirm this conclusion, we continued to perform a weight comparison of both models. Four different values were used for comparing Models 1a and 1b: WAIC, Pseudo-BMA without Bayesian bootstrap, Pseudo-BMA with Bayesian bootstrap, and Bayesian stacking (see Table S3). The Akaike weight was also used to rescale these values (McElreath, 2018). A total weight of 1 is partitioned among the two models, with the greater weight model implying better predictive accuracy on the data.

**Table S3:** Weight comparison between Models 1a and 1b

| | WAIC | Pseudo-BMA without Bayesian bootstrap | Pseudo-BMA with Bayesian bootstrap | Bayesian stacking |
|---|---|---|---|---|
| Model 1a | 0.7 | 0.73 | 0.65 | 0.69 |
| Model 1b | 0.30 | 0.27 | 0.35 | 0.31 |

As can be seen from Table S3, Model 1a outperforms Model 1b in predicting the available dataset, given that Model 1a's weight values double those of Model1b in all indicators.

Based on the statistical outcomes presented above, we determined to proceed with our study using Model 1a. Specifically, we only consider the non-linear relationships of *MigratIntention* with *NearbyMigratOpt* and *FarawayMigratOpt* in our subsequent models, but not their linear relationships. Not to say, adding more variables also makes the studied models complex beyond necessity and even raises more challenges for result interpretation.
