# Supplementary material for: Escaping from Air Pollution: Exploring the Psychological Mechanism behind the Emergence of Internal Migration Intention among Urban Residents
Source: Int J Environ Res Public Health. 2022 Sep 27;19(19):12233. doi: 10.3390/ijerph191912233 (PMC9566140; doi:10.3390/ijerph191912233)
Supplement: Supplementary file 1 [file ijerph-19-12233-s001.zip › Supplementary S2_Models convergence diagnoses.pdf]

Supplementary S2: Models' convergence diagnoses

The second Supplementary shows the visual convergence diagnoses of Models 1 and 2 using the Gelman plots and autocorrelation plots. In general, both models' convergence is confirmed by the Gelman plots and autocorrelation plots.

Model 1

Gelman plots of Model 1's parameters are shown in Figure S1. The *y*-axis of the Gelman plot illustrates the shrink factor (or Gelman factor), which is used to estimate the relative between the variance between Markov chains and the variance within chains. Meanwhile, the *x*-axis demonstrates the iteration order of the simulation. As can be seen, the shrink factors of all parameters drop rapidly to 1 during the warm-up iterations, hinting that there is no divergence among Markov chains. Therefore, the Markov property is held.

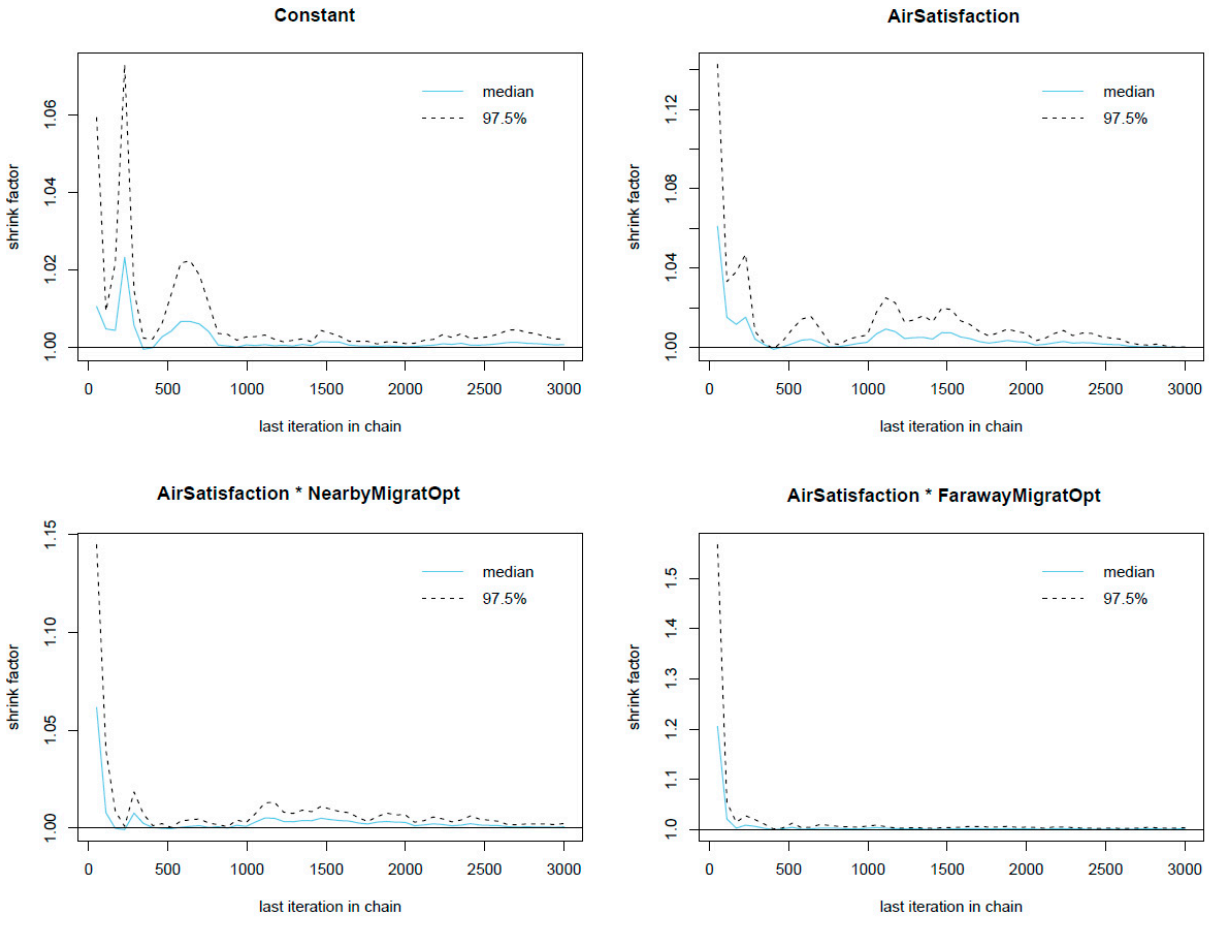

Figure S1: Gelman plots for Model 1's posterior parameters

Another further step to validate the convergence of Model 1's is to diagnose the Markov chains' autocorrelation levels visually (see Figure S2). The *x*-axes of the autocorrelation plots represent the number of Markov chains' lag, while the *y*-axes show each chain's average level of autocorrelation. Visually, the average autocorrelation level declines substantially before the fifth lag, inducing all parameters to acquire a great number of effective samples. The autocorrelation plots' demonstrations again confirm the convergence of Model 1's Markov chains.

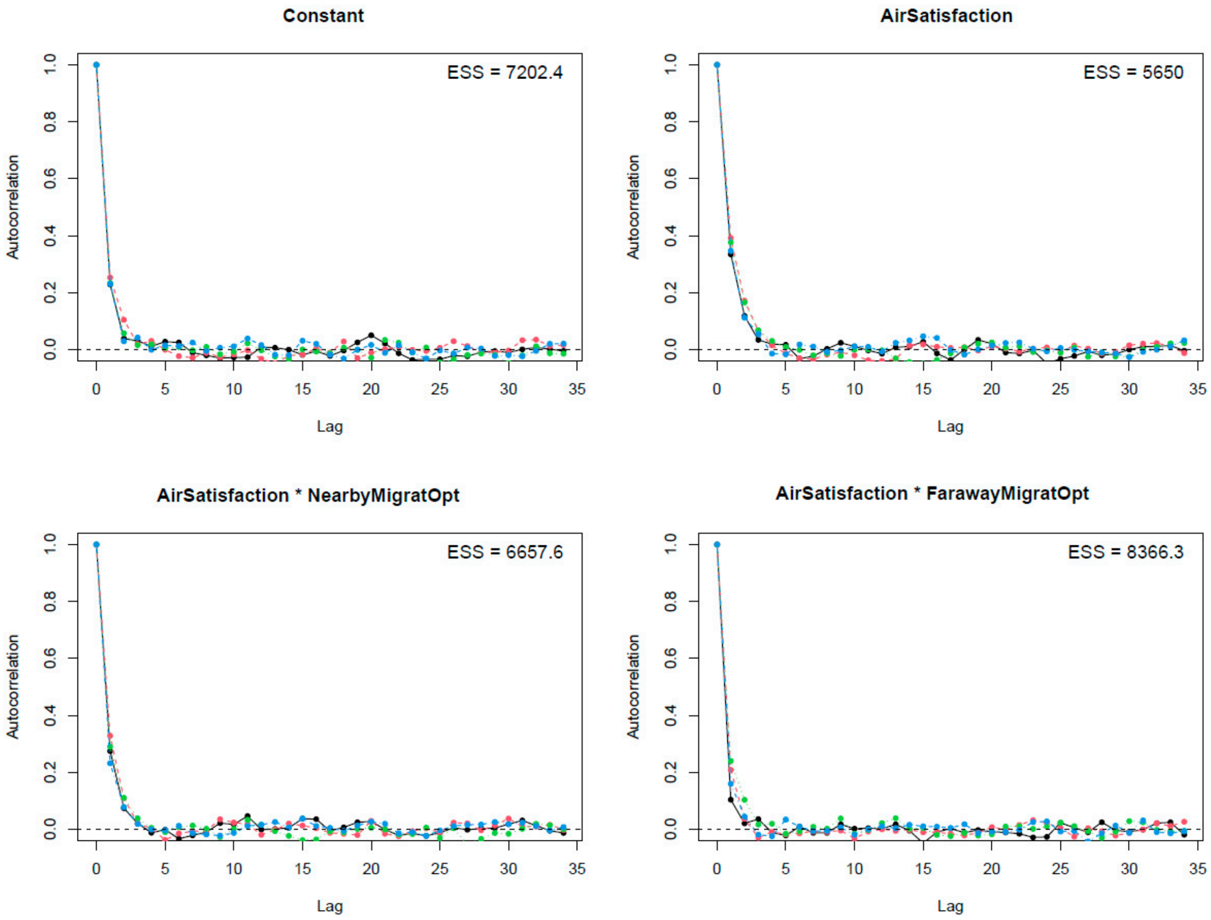

Figure S2: Autocorrelation plots for Model 1's posterior parameters

Model 2

Gelman plots of Model 2 show that the shrink factors of all parameters drop rapidly to 1 during the warm-up iterations, hinting that there is no divergence among Markov chains. Therefore, the Markov property is held.

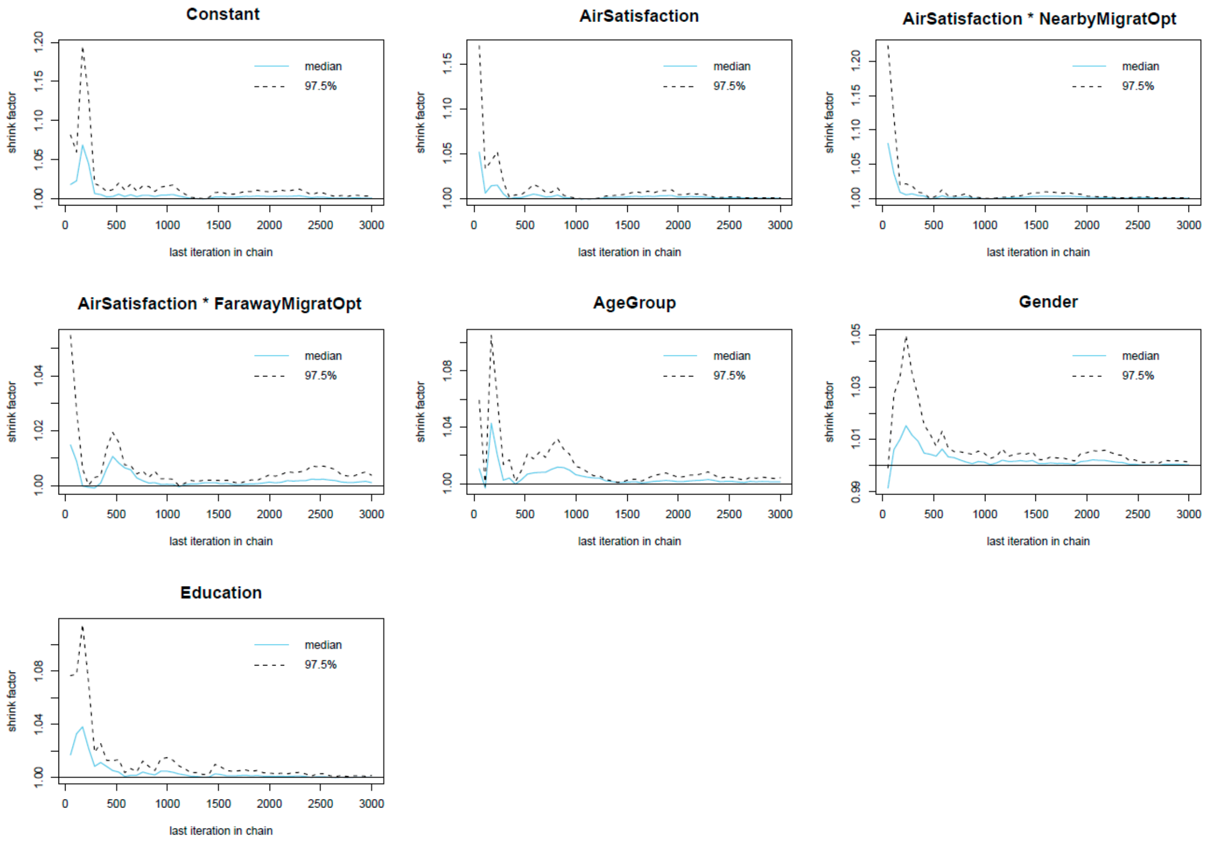

Figure S3: Gelman plots for Model 2’s posterior parameters

In Figure S4, the average autocorrelation levels of Model 2’s parameters decline substantially before the fifth lag, inducing all parameters to acquire a great number of effective samples. The autocorrelation plots’ demonstrations again confirm the convergence of Model 2’s Markov chains.

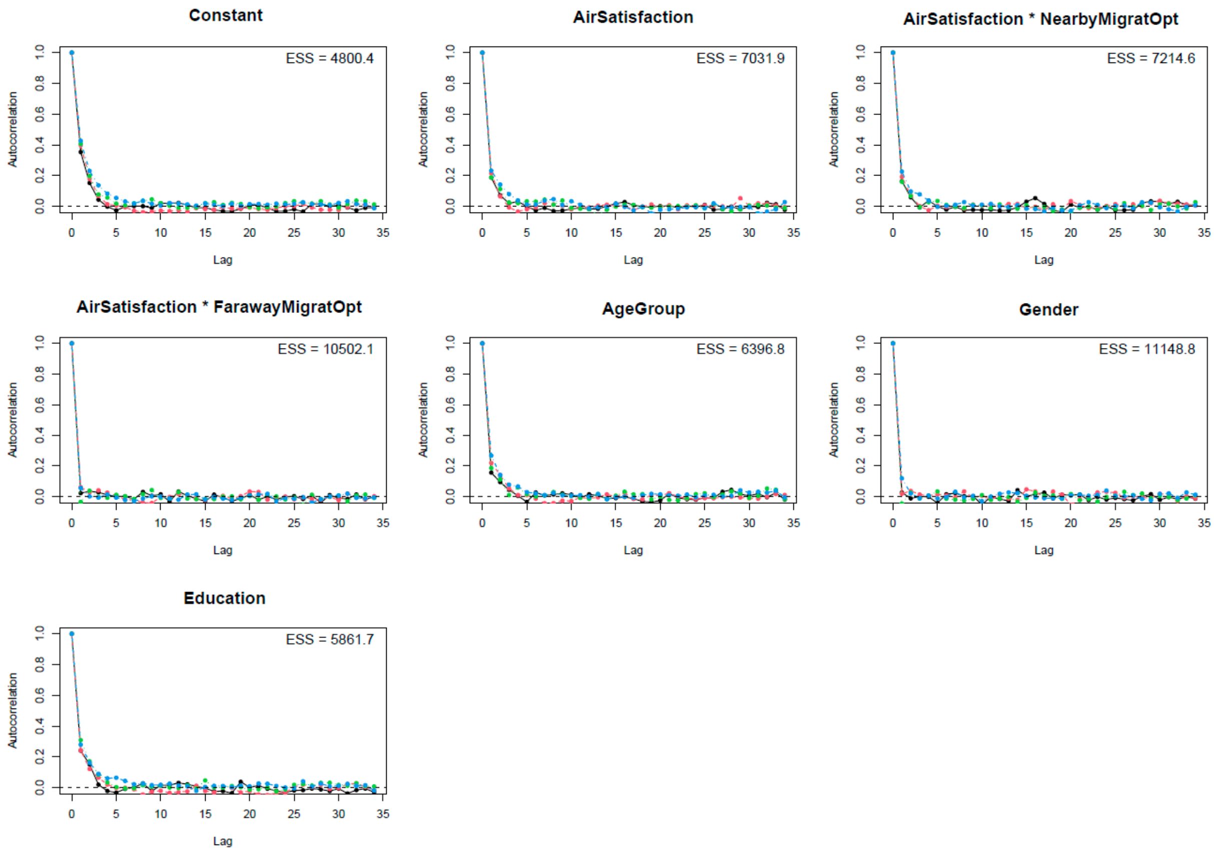

Figure S4: Autocorrelation plots for Model 2’s posterior parameters
